# Systematic Review of Cognitive Function in Euthymic Bipolar Disorder and Pre-Surgical Temporal Lobe Epilepsy

**DOI:** 10.3389/fpsyt.2017.00133

**Published:** 2017-08-09

**Authors:** Emmanuelle C. S. Bostock, Kenneth C. Kirkby, Michael I. Garry, Bruce V. M. Taylor

**Affiliations:** ^1^Psychology, School of Medicine, University of Tasmania, Hobart, TAS, Australia; ^2^Psychiatry, School of Medicine, University of Tasmania, Hobart, TAS, Australia; ^3^Menzies Institute for Medical Research, University of Tasmania, Hobart, TAS, Australia

**Keywords:** bipolar disorder, temporal lobe, focal seizures neuropsychology, cognition, epilepsy, systematic review

## Abstract

**Background:**

Bipolar disorder (BD) and temporal lobe epilepsy (TLE) overlap in domains including epidemiology, treatment response, shared neurotransmitter involvement and temporal lobe pathology. Comparison of cognitive function in both disorders may indicate temporal lobe mediated processes relevant to BD. This systematic review examines neuropsychological test profiles in euthymic bipolar disorder type I (BD-I) and pre-surgical TLE and compares experimental designs used.

**Methods:**

A search of PubMed, PsychINFO, and Scopus using Preferred Reporting Items for Systematic Reviews and Meta-Analyses guidelines was conducted. Inclusion criteria were comparison group or pre- to post-surgical patients; reported neuropsychological tests; participants aged 18–60 years. Fifty six studies met criteria: 27 BD-I; 29 TLE.

**Results:**

Deficits in BD-I compared to healthy controls (HC) were in executive function, attention span and verbal memory. Deficits in TLE compared to HC were in executive function and memory. In the pre- to post-surgical comparisons, verbal memory in left temporal lobe (LTL) and, less consistently, visuospatial memory in right temporal lobe (RTL) epilepsy declined following surgery. BD-I studies used comprehensive test batteries in well-defined euthymic patients compared to matched HC groups. TLE studies used convenience samples pre- to post-surgery, comparing LTL and RTL subgroups, few included comparisons to HC (5 studies). TLE studies typically examined a narrow range of known temporal lobe-mediated neuropsychological functions, particularly verbal and visuospatial memory.

**Conclusion:**

Both disorders exhibit deficits in executive function and verbal memory suggestive of both frontal and temporal lobe involvement. However, deficits in TLE are measured pre- to post-surgery and not controlled at baseline pre-surgery. Further research involving a head-to-head comparison of the two disorders on a broad range of neuropsychological tests is needed to clarify the nature and extent of cognitive deficits and potential overlaps.

## Introduction

Bipolar disorder type I (BD-I) is typically characterized by episodes of mania and depression with inter-episode euthymia. A number of impairments have been noted in the euthymic phase of the illness including social, occupational functioning, and cognitive deficits (1, 2).

Most studies have examined cognitive deficits in euthymic patients with BD-I compared to healthy controls (HC). Five meta-analyses have reported impairments in cognitive domains of executive functioning (3–7), verbal memory (3, 5–7), visuospatial memory (7), and attention (4, 6). One meta-analysis found no impairment of verbal memory and executive function in BD-I compared to HC (8). An individual patient data meta-analysis by Bourne et al. (9) reported impaired verbal memory and attention in euthymic BD-I patients relative to HC (9). The absence of an association between cognitive impairment and medication dose in euthymic BD-I patients suggest the effects of medication do not fully account for the cognitive impairments observed (4). These meta-analyses support the assumption that cognitive impairments exhibited in the euthymic phase are trait markers of the disorder.

An alternate research design is to compare and contrast cognitive deficits in BD-I to a reference condition which shares common features. For example, a meta-analysis comparing BD-I and schizophrenia reported more pronounced cognitive deficits in schizophrenia on measures of verbal fluency, verbal memory, executive function, visuospatial memory, mental speed, IQ, and concept formation (10). Similarly a single study comparing BD-I, obsessive compulsive disorder reported impaired verbal and episodic memory compared to HC (11). The BD-I group had greater impairments in learning word lists and delayed recall. These results suggest the importance of the temporal lobes in both disorders in the consolidation and retrieval of memories.

A single study compared BD-I with complex partial seizure disorder (12). It is noted that the classification of epilepsy syndromes has been subject to a number of iterations. In this review, we use the most commonly reported and widely understood term “temporal lobe epilepsy (TLE)” to incorporate the terms complex partial seizure disorder and focal seizures arising from the temporal lobes. The Jones et al. study reported greater impairment of executive function, attention and delayed verbal recall in the TLE group. These results should be interpreted with caution given the small and unequal sample sizes (BD-I *n* = 26, TLE *n* = 9). A case can be made for further exploration of similarities and differences between the neuropsychological test profiles seen in euthymic BD-I and interictal TLE. This is particularly so given the localizing pathology of TLE, which allows inferences to be made regarding the contribution of temporal lobe processes to the range of cognitive deficits reported in BD-I.

There are a number of established similarities between BD-I and TLE. These typically include a chronic course punctuated by episodic manifestations of mania and seizures, respectively. Other similarities include: the proposed involvement of kindling mechanisms (13); changes in neurotransmitters (excitatory amino acids, GABA, dopamine and serotonin), voltage-opened ion channels (sodium, calcium and potassium) and second messenger systems (G-proteins, phosphatidylinositol, protein kinase C, myristoylated alanine-rich C kinase substrate), and treatment response to antiepileptic medications in both disorders (14).

In addition, episodes in both disorders commonly feature sensory, perceptual, cognitive, and affective changes (15) including depression (16). Epidemiological studies have shown that the proportion of BD-I among people with epilepsy is more than twice as high as in the general population (17) and that mania is more common in patients with TLE than in the general population (18). BD-I is also associated with comorbid epilepsy but not parental epilepsy (19). Episodes of mania in BD-I and seizures in TLE share precipitating factors including stress, sleep reduction and antidepressant medications (20).

The temporal lobes have also been the subject of neuroimaging research in both disorders. In BD-I many studies have investigated correlates of the disorder to specific brain regions. Meta-analyses of magnetic resonance imaging (MRI) studies have reported that BD-I is associated with right lateral ventricular enlargement (21) and an enlarged left amygdala (22). However, studies of temporal lobe size are inconsistent; with reported increases (23), reductions (24) or no differences (25–28) likely reflecting the difficulties of defining and measuring the volume of individual cerebral lobes on MRI.

In TLE, MRI studies have reported structural brain abnormalities in the hippocampus, entorhinal cortex (29), thalamus (30), and fornix (31). On voxel-based morphometry, TLE is associated with gray matter pathology in the hippocampus, cingulum, thalamus, and frontal lobes. White matter reductions ipsilateral to the seizure focus were also found in the temporopolar, entorhinal, and perirhinal areas (32). Typically, TLE originates unilaterally from the medial temporal lobe; they may, however, be propagated from other regions which project to limbic areas (33).

Given these potential diffuse structural abnormalities seen in patients with TLE, it could be expected that neuropsychological deficits may not be limited to tasks involving temporal lobe function. Patients with TLE display deficits in memory, general intelligence, language, executive function, and motor speed relative to HC (34, 35). Deficits in verbal memory, language, and psychomotor speed may be affected by factors such as age of onset of epilepsy, general intelligence, the number and dose of antiepileptic medications used, and seizure frequency (35).

The literature describing clinical features, imaging findings, and neuropsychological test profiles is largely in a separate corpus for BD-I and for TLE with only one small study directly comparing the two (12).

The current structured review brings these two bodies of work together in a comparison of the neurocognition literature findings of the two conditions side-by-side. The aim is to determine whether and to what extent the cognitive impairments seen in euthymic BD-I are mirrored by those attributed to a pathology primarily affecting the temporal lobes, that is TLE.

## Method

This systematic review was conducted in accordance with the Preferred Reporting Items for Systematic Reviews and Meta-Analyses (PRISMA) guidelines (36).

*Inclusion criteria*: (a) controlled comparison, (b) patients diagnosed with BD-I as assessed by a recognized criterion-based diagnostic system, (c) BD-I patients rated as euthymic, defined by their scores on a rating scale (<8 HamD, <8 YMRS); patients with unilateral TLE, from any cause, with diagnosis confirmed in a pre-surgical workup, (d) at least one neuropsychological test, (e) adult participants (18–60 years), (f) in the case of more than one article by the same authors results were not identified as being from the same sample, (f) articles published 1980 or later.

### Identification of Studies

A comprehensive search of the electronic databases PubMed, PsychINFO, and Scopus for peer-reviewed articles published in English was conducted in the last week of May 2016. Search terms were grouped as follows: bipolar, manic depress*, baseline, asympt*, remit*, stable (group 1); or epilep*, seizure, presurg*, temporal, focal, partial, complex, interictal (group 2) and WAIS, Wechsler, trail making, continuous performance, stroop, digit span, verbal learning, rey, working memory, benton, card sort*, verbal fluency, RAVLT, CAVLT, tower of London (group 3). These search terms were combined as follows: group 1 AND group 3 for BD-I and group 2 AND group 3 for TLE.

### Data Extraction

**Figure 1:**
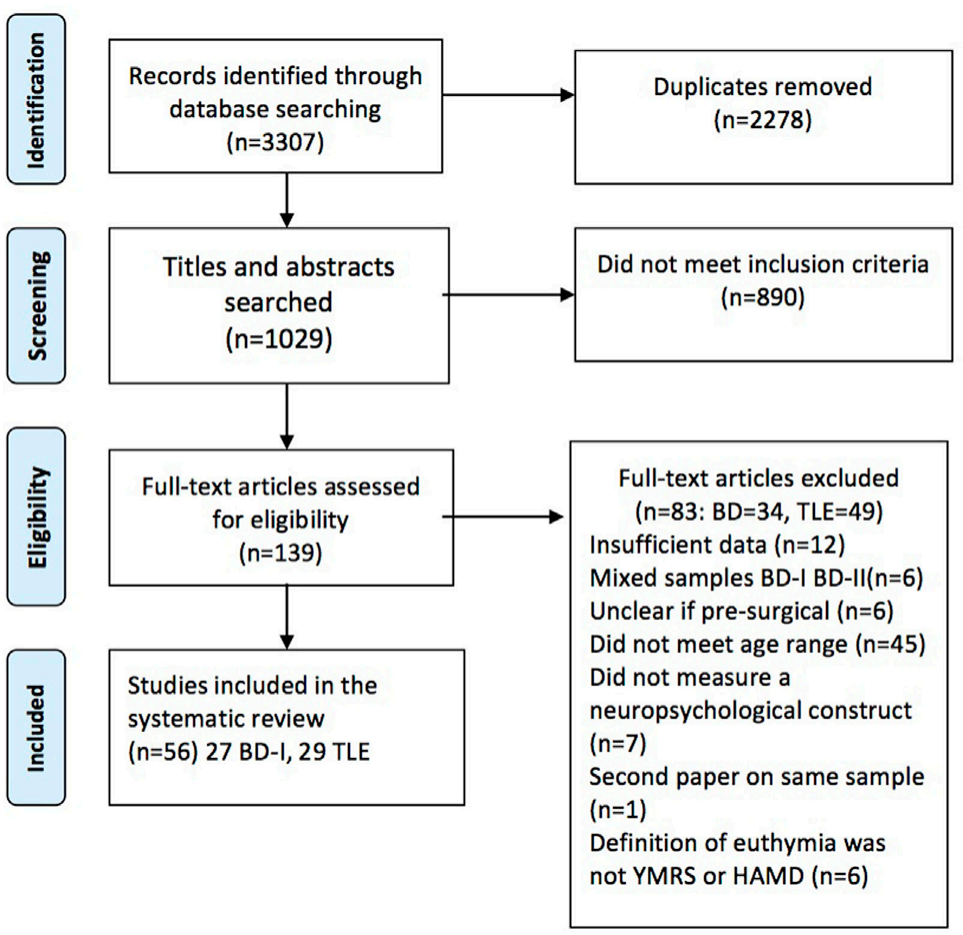
Preferred Reporting Items for Systematic Reviews and Meta-Analyses flow diagram of included studies.

The abstracts located from the search strategy were entered into EndNoteX7. The PRISMA flowchart (Figure 1) sets out the steps in screening conducted by author EB. After the screening of titles and abstracts the remaining 139 studies were examined in full text, using a purpose built coding sheet to assess whether they met inclusion criteria. This process resulted in the study sample of 56, of which 27 related to BD-I and 29 to TLE.

## Results

The search strategy identified an initial 3,307 articles of which 56 met inclusion criteria, 27 related to BD-I and 29 to pre-surgical TLE. Studies of BD-I are summarized in Table 1 and of TLE in Table 2 detailing authors, sample sizes, medication (BD-I only as this was not recorded in the TLE samples), neuropsychological test parameters, and summary results.

**Table 1:** Summary of included studies on euthymic bipolar disorder.

Reference	*n*	Medications bipolar sample	Neuropsychological test parameters	Results
Altshuler et al. (37)	BD 40	Li 25, AC 12, AD 4, AP 6, BZD 3	WAIS-R block design, vocab; TMT, WCST, Stroop; VFT, CVLT; ROCFT; Star mirror tracing Task, PR	BD impaired verbal memory and executive functioning
	SZ 20			
	HC 22			

Bas et al. (38)	BD 60	Li 39, AP 37, LTG 9, VPA 21	Stroop, TMT A and B; WMS-R visual reproduction; RAVLT	BD impaired on RAVLT
	HC 41			

Bora et al. (39)	BD 514	Li 63.2%, VPA 47.6%, AP 47.2%, AD 5.7%	Stroop. WCST	Deficits exist in subgroups who have severe and global cognitive deficits
	BD-II 42			
	HC 416			

Chang et al. (40)	BD 23	BD Li 13, AP 15, LTG 7, VPA 9	WAIS-R block design and vocab; CVLT, VFT	All NS
	BD-II 23			
	HC 23			

Chou et al. (41)	BD 23	AP 13, VPA 19	The Color Trails Test, WCST; WMS-III – Word Lists Test, Face Test; Go/No-GO, Test for Attentional Performance	BD impaired faces memory and WCST
	HC 33			

Deckersbach et al. (42)	BD 25	Li 11, AC 9, AD 9, AP 3	ROCFT	Immediate recall BD impaired, copy and recognition preserved
	HC 25			

Deckersbach et al. (11)	BD 30	Li 13, AC 11, AD 8, AP 4	CVLT	CVLT BD impaired learning and delayed free recall
	Obsessive compulsive disorder 30			
	HC 30			

Dell’Osso et al. (43)	BD 15	BD Mono 2, Poly 12	N-Back	NS HC and BD
	BD-II13			
	HC 27			

Dittmann et al. (44)	BD 65	BD Li 27, AD 10, AP 33, CBZ 4, VPA 28	TMT; HAWIE-R; WAIS-III letter number sequencing; RBANS	Psychomotor speed, working and delayed memory, verbal learning, executive functioning BD impaired
	BD-II 38			
	HC 62			

Dixon et al. (45)	BD manic 15 dep 15, eu 15, HC 30	Eu Li 15, AD 8, AP 10, AC 2	WAIS-R Vocab; Stroop; FAS; Hayling sentence completion test; Cognitive Estimates Test	VF total responses, Hayling response initiation latency, error score and using strategy, stroop EuBD impaired

Doganavs¸argil-Baysal et al. (46)	BD 60	Li 11, MS + AP 12, 2MS 12, VPA 6	WCST, TMT, Stroop; RAVLT; Cancelation test	Significant differences on all measures between BD and HC
	HC 20			

Doganavs¸argil-Baysal et al. (46)	BD 54	Li 10, MS + AP 27, 2MS 11, VPA 5	WCST; RAVLT	Both measures BD < HC
	HC 18			

Doruk et al. (47)	Manic 20	Unknown	Stroop; Serial Digit Learning Test; RAVLT; Cancelation Test	NS HC and EuBD
	Dep10			
	Eu 21			

Fistikci et al. (48)	BD 25	Li 25	WCST; Montreal Cognitive Assessment	NS HC and BD
	HC 25			

Frangou et al. (49)	BD 10	Li 5, AC 3	WCST; Hayling sentence completion	WCST BD and offspring made more errors
	Un offspring 15			
	HC 43			

Hsiao et al. (50)	BD 30	VPA 29	WAIS-III digit symbol; TMT; WMS-III	Verbal memory, working memory, psychomotor speed, executive function BD impaired
	BD-II 37			
	HC 22			

Martino et al. (51)	BD 48	AD 16, AP 28, BZD 23, MS 48	WAIS vocab, digit span; TMT, WCST, IGT; Memory battery of Signoret	Verbal memory, attention and executive functions impaired
	BD-II 37			
	HC 34			

Muralidharan et al. (52)	BD 72	AP 27, Li 34, VPA 38	TMT, Stroop; CANTAB; CVLT; WMS-III LNS	P on VPA more working memory deficits than Li or HC
	HC 40			

Muralidharan et al. (53)	BD 68	AP 51, Li 32, MS + AP 50, VPA 32	TMT, Stroop; CANTAB; CVLT; WMS-III letter number sequencing	Verbal and visuospatial memory, working memory and executive function BD impaired.
	HC 38			

Normala et al. (54)	BD 40	AP 6, MS 11, MS + AD 1, MS + AP22	WAIS Digit span; TMT; Verbal Fluency	Executive and attention functioning BD impaired
	HC 40			

Pattanayak et al. (55)	BD 30	AP 5, LTG 2, Li 21, VPA 11	VIQ; TMT, Stroop; N-Back; Postgraduate Institute Memory Scale	Attention, information processing speed, executive function, verbal memory BD impaired
	HC 20			

Radwan (56)	BD 30	Unknown	WAIS; WCST; WMS; CPT	All BD impaired
	HC 30			

Sepede et al. (57)	BD 24	AD 10, AP 15, BZD 8, Li 3, MS 9	CPT	Sustained attention impaired BD and rels
	Unaffected rels 33			
	HC 24			

Trivedi et al. (58)	BD 15	CBZ 1, Li 8, VPA 6	WCST; SWMT; CPT	Executive function BD impaired
	HC 15			

Trivedi et al. (59)	BD 15	Unknown	WCST; SWMT; CPT	Executive function BD impaired
	SZ 15, HC 15			

Yates et al. (60)	BD dep 34	AD 9, AP 18, BZD 9, MS 29	WAIS-III	Verbal measure BD impaired
	BDEu 31, HC 34			

Zubieta et al. (61)	BD 15	AP 3, CBZ 2, Li 7, VPA 12	WAIS-R: digit span; WCST, Stroop; WMS-R; verbal fluency; test of variable attention	Verbal learning, executive function, motor coordination and sequential memory BD impaired. NS verbal fluency or attention
	HC 15			

*BD, bipolar; BD-II, bipolar II; HC, healthy controls; SZ, Schizophrenia; Li, lithium; AC, anticonvulsant; AD, antidepressant; AP, antipsychotics; BZD, benzodiazepine; CBZ, carbamazepine; LTG, lamotrigine; MS, mood stabilizer; VPA, valproate; Mono, monotherapy; Poly, polytherapy; AMIPB, Adult Memory and Information Processing Battery; BVRT, Benton Visual Retention Test; VLMT, Verbaler Lern- und Merkfähigkeitstest; DCS-R, Diagnosticum für Cerebralschädigung – II; NAART, North American Adult Reading Test; WRAT, Wide Range Achievement Test; WAIS, Wechsler Adult Intelligence Scale; CPT, Continuous Performance Test; CVLT, California Verbal Learning Test; RAVLT, Rey Auditory Verbal Learning Test; WCST, Wisconsin Card Sorting Test*.

**Table 2:** Summary of included studies on pre-surgical temporal lobe epilepsy (TLE).

Reference	*n*	Neuropsychological test parameters	Results
Baxendale and Thompson (62)	Right temporal lobe (RTL) 133	WAIS PIQ, VIQ; AMIPB	Verbal memory decline post-surg left temporal lobe (LTL)
	LTL 157		

Baxendale et al. (63)	RTL 146 LTL 177	WAIS PIQ, VIQ; AMIPB; Birt Memory and Information Processing Battery	RTL and LTL at equal risk of post-surg decline

Berenbaum et al. (64)	LTL 57	WAIS Digit Span; CVLT	CVLT decline post-surg

Bjørnaes et al. (65)	RTL 50	WAIS Digit Span; Benton Visual Retention Test (BVRT); Design Learning and Retention Test; Verbal List Learning and Retention; Tactual Performance Test	Improvement at 2-year follow up post-surg
	LTL 41		

Chelune et al. (66)	RTL 19	WAIS-R VIQ, PIQ; WMS-R, RAVLT; COWAT, Halstead-Wepman Aphasia Screening Exam, BNT, Speech Sounds Perception Test; Hooper Visual Organization Test, Seashore Rhythm Test	LTL decline post-surg
	LTL 23		

Chiaravalloti et al. (67)	RTL 16	WMS-III Faces Subtest; Graduate Hospital Facial Memory Test	RTL < LTL both pre- and post-surg
	LTL 10		

Chiaravalloti (68)	RTL 42	CVLT; Graduate Hospital Facial Memory Test	Verbal memory post-surg decline LTL, RTL improved. Visuospatial memory post-surg decline RTL, LTL improvement
	LTL 28		

Fernandes et al. (69)	RTL 23	WAIS-R Block design, Vocabulary; WMS-R; RAVLT	Cognitive scores post-surg decline low pre-surg scores. Non-verbal memory post-surg RTL decline, verbal and visuospatial memory LTL decline
	LTL 24		
	healthy controls (HC) 28		

Giovagnoli et al. (70)	RTL 12	Raven’s Colored Progressive Matrices; Attentive Matrices; Verbal Selective Reminding Procedure, Story Recall, Verbal Memory Distractor Test; Visual Selective Reminding Procedure, ROCFT, Visual Memory Distractor Test	Verbal memory pre- and post-surgery LTL impaired relative to HC. Visual deficits present in both groups relative to HC
	LTL 12		
	HC 36		

Gleissner et al. (71)	RTL 63	VLMT; DCR-S	Verbal memory LTL decline
	LTL 52		

Glosser et al. (72)	RTL 13	Boston Naming Test; CVLT Benton Facial Recognition, Graduate Hospital Facial Memory	Recognition of familiar faces and learning new faces RTL impaired. Names of familiar faces LTL impaired
	LTL 8		
	HC 10		

Helmstaedter et al. (73)	LTL 47	Verbal Learning and Memory Test; RAVLT	Delayed recall and recognition post-surg improvement

Hermann and Wyler (74)	RTL 14	COWAT; Token Test	Language tests pre-surg LTL deficit
	LTL 15		

Hermann et al. (75)	RTL 31	CVLT	Verbal memory post-surg RTL increased
	LTL 26		

Hermann et al. (76)	RTL 26	WAIS-R Digit Span; Multilingual Aphasia Examination Visual Naming Test; CVLT	CVLT post-surg decline LTL
	LTL 36		

Köylü et al. (77)	RTL 12	List learning task	LTL post-surg decline
	LTL 14		

Lee et al. (78)	LTL 38	RAVLT; ROCFT	Memory post-surg decline

Loring et al. (79)	RTL 13	Selective Reminding Test, Serial Digit Learning, ROCFT, Form Sequence Learning	Complex figure RTL impaired. Verbal memory decline LTL
	LTL 16		

Malikova et al. (80)	RTL 11	WAIS-R; WMS-R; Verbal Fluency Test	FSIQ, global and verbal memory, attention, and working memory all improved post-surg
	LTL 26		

Morino et al. (81)	RTL 31	WMS-R; Miyake Verbal Retention Test; BVRT	Memory RTL improved post-surg. Verbal memory LTL post-surg improved
	LTL 31		

Quigg et al. (82)	RTL 16	TMT; BNT; CVLT; WMS-R Logical Memory Scale	BNT and CVLT LTL decreased post-surgery. Language and verbal memory LTL increased. TMT increased
	LTL 14		

Seidenberg et al. (83)	RTL 30	CVLT	Free recall LTL decline post-surgery
	LTL 46		

Selwa et al. (84)	RTL 14	WAIS-R; WMS	FSIQ, Logical Memory RTL improved post-surg. Verbal memory decline LTL post-surg
	LTL 17		
	Non-surgical TLE 28		

Shamim et al. (85)	RTL 14	WAIS-III; WMS-III	Verbal memory deficit post-surg LTL
	LTL 16		

Stretton et al. (86)	RTL 17	WAIS-III Digit Span; Gesture Span Task; Motor Sequences Task; Dot-Back Paradigm	Working memory pre-surg RTL and LTL worse than HC. WM improved post-surg LTL
	LTL 16		
	HC 15		

Tisser et al. (87)	RTL 10	WAIS-R; WCST	WCST RTL and LTL impaired, improved post-surg
	LTL 15		
	HC 22		

Trenerry and Jack (88)	RTL 34	WAIS-R; WCST	The WCST is not useful for lateralizing seizure onset in TLE
	LTL 34		

Trenerry et al. (89)	RTL 36	WAIS-R; WMS-R; RAVLT; Visual Spatial Learning Test	Verbal and visual memory LTL improved post-surg
	LTL 44		

von Rhein et al. (90)	RTL 20	VLMT; DCS-R; BNT; Token Test	Verbal Memory impaired post-surgery. Naming decline post-surgery LTL
	LTL 32		

*Tests of executive function: WCST, FAS, TMT-B, Stroop, Hayling Sentence Completion Test, CANTAB: Intra Extra Dimensional Set Shifting, CANTAB: Stockings Problem; Tests of Verbal Memory—CVLT, RAVLT, WAIS Vocab, WMS-R: Logical Memory, WMS-R: Verbal Paired Associates, Token Test, VLMT: Verbaler Lern- und Merkfähigkeitstest; Tests of non-verbal memory- ROCFT, WMS: Visual reproduction, WMS-R: Design Memory, WMS-R Visual Reproduction, WMS-III: Face Test, CANTAB: Spatial recognition memory, CANTAB: Pattern Recognition Memory, CANTAB: Paired Associates Learning; Attention span WAIS: Digit Span, Adult Memory and Information Processing Battery (AMIPB); sustained attention CPT; working memory SWMT, N-Back, WAIS: Letter Number Sequencing, WMS-III: Letter Number Sequencing, CANTAB: Spatial Working Memory*.

As demonstrated in Table 1, 25 of 27 studies compared patients with BD-I (*n* = 1,398) to HC (*n* = 1,142). The remaining studies compared manic, euthymic, and depressed groups. The most commonly reported impairments in BD-I were in executive function, attention span, and verbal memory. No studies found enhanced neuropsychological function in euthymic BD-I.

As shown in Table 2, in the epilepsy literature, all of the TLE studies compared neuropsychological test performance pre- to post-surgery. Only 5 of the 29 studies compared pre-surgical TLE (*n* = 150) to HC (*n* = 111); these studies found significant impairments in TLE compared to HC on tests of memory and executive function.

In 26 of the TLE studies, the samples were divided by laterality of seizure focus with the primary pathology affecting the right temporal lobe (RTL *n* = 846) or the left temporal lobe (LTL *n* = 1,068). In pre-surgical TLE, the direct comparison of LTL and RTL groups indicated that the LTL group showed impaired verbal memory and the RTL group, less consistently, impaired visuospatial memory.

The most common impairment observed in TLE related to surgery was in verbal memory. This finding was also associated with laterality, 24 studies reporting decline in verbal memory from pre- to post-surgery in LTL patients, whereas in RTL there was a less consistent decline in visuospatial memory. No significant differences in attention were found for laterality or pre- to post-surgery in TLE.

Table 3 shows the results of individual neuropsychological tests that were reported in more than one study, for BD-I and TLE. Of note, the total number of neuropsychological tests used across all studies differed between BD-I (27) and TLE (11); this was not evenly distributed across cognitive domains. The number of studies where any executive function instrument was administered in TLE was only 4 (2 WCST, 2 COWAT) of 29 compared to 21 of 27 in BD-I. One study compared RTL, LTL and HC and showed that all patients were impaired on the WCST relative to HC (87). The number of studies where any verbal memory instrument was administered was 26 of 29 in TLE compared to 14 of 27 in BD-I. The comparable figures for visuospatial memory instruments were 25 of 29 in TLE and 11 of 27 in BD-I. Similarly, only 6 studies measured attention in TLE compared to 17 studies in BD-I. Thus, while both fields have seen sustained research activity in identifying neuropsychological deficits, the focus of inquiry in TLE has been on verbal and visuospatial memory and in BD-I executive function.

**Table 3:** Neuropsychological test findings summary table (for tests used more than once) in studies of bipolar disorder (BD) and temporal lobe epilepsy (TLE).

Measure	BD		TLE		Pre- to post- surgical		Laterality effects
	Use in studies, number of participants BD	BD < HC sig	Use in studies, number of participants TLE	sig	↑	↓	
**Executive function**							
WCST	12 studies	11	2 studies	1	↑1		
	BD *n* = 859, healthy controls (HC) *n* = 676		Right temporal lobe (RTL) *n* = 44, left temporal lobe (LTL) *n* = 49, HC *n* = 22				
COWAT(FAS)	5 studies	2	2 studies	2	↑1		Higher score LTL group pre- assoc. with greater impairment post-surg
			RTL *n* = 33, LTL *n* = 38				
	BD *n* = 133, HC *n* = 130						
TMT-B	9 studies	7					
	BD *n* = 513, HC = 339						
Stroop	10 studies	7					
	BD *n* = 895, HC = 642						
Hayling Sentence Completion Test	2 studies	2					
	BD *n* = 25, HC *n* = 73						
CANTAB: Intra Extra Dimensional Set Shifting	2 studies	2					
	BD *n* = 140, HC *n* = 78						
CANTAB: Stockings Problem	2 studies	2					
	BD *n* = 140, HC *n* = 78						

**Verbal memory**							
CVLT	5 studies	3	7 studies	6		↓6	Post-surg LTL decline 6
	BD *n* = 233, HC *n* = 153		RTL *n* = 158, LTL *n* = 215, HC *n* = 10				RTL > LTL 1
RAVLT	3 studies	2	5 studies	5		↓5	Post-surg LTL decline 5
	BD *n* = 141, HC = 79		RTL *n* = 78, LTL *n* = 176, HC *n* = 28				
Verbal comprehension: WAIS Vocab	4 studies	0					
	BD *n* = 187, HC *n* = 173						
WMS-R: Logical Memory			6 studies	4	↑3	↓1	LTL < RTL 1
			RTL *n* = 206, LTL *n* = 256, HC *n* = 28				
WMS-R: Verbal Paired Associates			4 studies	4	↑2	↓1	LTL < RTL 1
			RTL *n* = 119, LTL *n* = 151, HC *n* = 28				
Token Test			2 studies	1	↑1		Post-surg LTL improved 1
			RTL *n* = 34, LTL *n* = 47				
VLMT: Verbaler Lern- und Merkfähigkeitstest			2 studies	2		↓2	
			RTL *n* = 83, LTL *n* = 82				

**Visuospatial memory**							
ROCFT	2 studies	0	3 studies	2	↑1		LTL < RTL 1
	BD *n* = 65, HC *n* = 47		RTL *n* = 25, LTL *n* = 62, HC *n* = 36				
WMS: Visual reproduction	3 studies	0	2 studies	0			
	BD *n* = 105, HC *n* = 78		RTL *n* = 27, LTL *n* = 29				
CANTAB: Spatial recognition memory	2 studies	2					
	BD *n* = 140, HC *n* = 78						
CANTAB: Pattern Recognition Memory	2 studies	2					
	BD *n* = 140, HC *n* = 78						
CANTAB: Paired Associates Learning	2 studies	2					
	BD *n* = 140, HC *n* = 78						
WMS-R: Design Memory			4 studies	2	↑1	↓1	RTL decline 1
			RTL *n* = 78, LTL *n* = 93, HC *n* = 28				
WMS-R Visual Reproduction			5 studies	2	↑1	↓1	RTL decline 1
			RTL *n* = 241, LTL *n* = 484, HC *n* = 28				
WMS-III: Face Test			1 study	1		↓1	RTL decline 1
			RTL *n* = 16, LTL *n* = 10				
Graduate Hospital Facial Memory			3 studies	3	↑1		RTL < LTL 2
			RTL *n* = 71, LTL *n* = 46, HC *n* = 10				
Benton Visual Retention			2 studies	0			
			RTL *n* = 31, LTL *n* = 72				
Diagnosticum für Cerebralschädigung			3 studies	2		↓1	
			RTL *n* = 83, LTL *n* = 253				

**Spatial ability**							
WAIS: Block design	4 studies	0					
	BD *n* = 124, HC *n* = 109						

**Attention span**							
WAIS: Digit Span	3 studies	2	4 studies	0			
	BD *n* = 103, HC *n* = 89		RTL *n* = 93, LTL *n* = 150, HC *n* = 15				
TMT-A (also processing speed)	10 studies	8					
	BD *n* = 513, HC = 339						
WAIS: Digit Symbol (also processing speed)	3 studies	3					
	BD *n* = 91, HC = 86						
CANTAB: Rapid Visual Information	2 studies	2					
	BD *n* = 140, HC *n* = 78						
Adult Memory and Information Processing Battery (AMIPB)			2 studies	2	↑1	↓1	
			RTL *n* = 279, LTL *n* = 334				

**Sustained attention**							
CPT	4 studies	2					
	BD *n* = 84, HC *n* = 84						

**Working memory**							
SWMT	2 studies	0					
	BD *n* = 30, HC *n* = 30						
N-Back	2 studies	0					
	BD *n* = 45, HC *n* = 47						
WAIS: Letter Number Sequencing	3 studies	3					
	BD *n* = 126, HC *n* = 126						
WMS-III: Letter Number Sequencing	2 studies	3					
	BD *n* = 140, HC *n* = 78						
CANTAB: Spatial Working Memory	2 studies	2					
	BD *n* = 140, HC *n* = 78						

↑ *refers to an increase*, ↓ *a decrease from pre- to post-surgery, sig refers to the number of studies with a significant result, laterality comments only significant main effects or interactions are discussed*.

Notwithstanding the differences in the intensity and focus of neuropsychological testing in both conditions, consistent results emerging from this study emphasize deficits in verbal memory, which have been reported in the majority of studies that have examined this area in both BD-I and TLE.

## Discussion

To our knowledge, this is the first systematic review directly comparing the literature on cognitive function in BD-I and TLE. Consistent with the meta-analyses of cognition in euthymic BD-I, our review showed impairments on a wide range of cognitive measures. In the individual studies reviewed, the most commonly reported impairments in BD-I were in executive function, attention span, and verbal memory. The impairments of executive function in patients with BD-I may be suggestive of an underlying dysfunction in the prefrontal cortex (3).

In TLE studies, a decline in pre- to post-surgery in verbal memory was commonly reported in patients with seizures originating from the LTL, and less consistently, in visuospatial memory in patients with RTL epilepsy. Functional MRI studies have revealed that the right hemisphere is associated with spatial memory (91). In our sample, executive function was not widely examined in patients with epilepsy; however, an impairment was found. For executive function, two instruments were employed across four studies, three of which showed a difference between pre- to post-surgery scores and one study found impaired performance in TLE relative to HC participants.

In keeping with our results, meta-analyses of memory function pre- to post-surgery reported that, following a resection of the LTL, a clear decline in verbal memory is observed, an effect that is particularly salient for immediate verbal recall. However, the pattern of impairment following partial resection of the RTL showed a trend for improvement on tasks of non-verbal memory (92). This suggests that memory impairments are state markers affected by seizures and abnormalities in the temporal lobes. Other factors that affect cognitive performance in TLE are the chronicity of the condition, older age, lower intellect, and greater abnormalities shown on imaging (92). Another meta-analysis found that the evidence regarding post-surgery outcome on visuospatial memory following right anterior temporal lobectomy was less clear (93).

In TLE, it is unclear whether frontal lobe impairments shown on executive function measures are a product of temporal lobe involvement or are a side effect of the propagation of epileptic activity from the epileptic zone (94). Other evidence has suggested that the prefrontal cortex, in particular the orbitofrontal cortex, is influenced by ictal discharges from the mesial temporal lobe (95). Some studies have shown that the temporal neocortex is implicated in executive function implying that a frontotemporal network is used for processing information (96).

This review emphasizes that prior research on cognitive impairments in the fields of BD-I and TLE has employed methodologies that reflect different research questions. The BD-I literature predominantly examines cognition as a characteristic of the disorder itself, on a par with symptoms and potentially amenable to therapeutic intervention. The TLE literature is concerned with the effects of ablative surgery that aims to remove seizure foci but may consequently also directly affect healthy brain tracts. It addresses whether cognitive function improves or declines subsequent to surgery and the moderating effects of laterality.

The majority of BD-I studies compare euthymic patients with HC, whereas in the epilepsy studies, patients act as their own controls in relation to surgical intervention and laterality. In addition, given that the BD-I studies are interested in trait markers in the euthymic phase, they routinely report the quantitative differences between patients and HC, rendering the results suitable for incorporation in meta-analytic studies. The TLE literature has focused on statistically significant differences pre- to post-surgery and has not reported information in a form suitable for meta-analysis of pre-surgical cognitive functioning. Therefore, we have employed a systematic review methodology to examine and compare the profile of cognitive deficits in the two conditions. As indicated in the Section “Results,” the focus of inquiry of the neuropsychological tests employed in the BD-I studies is predominantly on frontal lobe functions and in TLE on verbal and visuospatial memory.

There are significant differences in the experimental designs examining cognition in the two patient groups. In BD-I studies, cognition is tested broadly with a wide range of measures, with a HC comparison group and statistical control for medication use. This allows for observed deficits to be interpreted as trait markers of BD-I. This is further supported by familial studies that show similar patterns of impairments among family members and patients (89). By contrast, studies assessing cognitive performance in pre-surgical patients with epilepsy do so generally without HC. Only five studies included HC (*n* = 111), consequently the findings have limited depth compared to those in the BD-I literature (*n* = 1,142). The pre-surgical neuropsychological workup consists mostly of tests of memory function, which is not surprising given that the surgery involves removing parts of or the whole temporal lobe.

In general, the studies on BD-I report the types of medications taken by patients at the time of testing (as shown in Table 1), which may have impacted the results. By contrast, in the epilepsy samples, it was typically not reported whether patients were receiving medication at the time of testing. A study that examined the effects of atypical antipsychotics on cognition in euthymic BD-I patients found that untreated patients showed better performance than those taking medication (93). Many patients with BD-I are treated with anticonvulsants that may worsen or enhance cognition (97). Of the total sample of 884 patients with BD-I included in the systematic review, 299 were taking lithium at the time of testing. A review of the effects of lithium on cognition found that impairments on tasks of psychomotor speed and verbal memory were present, whereas no effect was found on visuospatial ability or attention (97). Thus, cognitive performance may be impaired in various ways by different medications. A recent randomized crossover study examined the effects of methylene blue on cognition and mood-related symptoms in euthymic BD-I and BD-II. Neither low (15 mg) control doses nor high active doses (195 mg) had a significant effect on cognition (98). In rats, methylene blue prevents methylmalonate-induced seizures and oxidative damage in the striatum (99) providing interesting leads for future research into the overlaps between BD and epilepsy.

While this paper has provided an overview of the literature, it is subject to a number of limitations. One such factor is the differences of experimental designs in BD-I and TLE, which meant that a head-to-head meta-analytic comparison was not feasible. As discussed previously, the effect of medication was not uniformly controlled in BD-I and was either not reported or not systematically recorded in the epilepsy samples. In order to determine whether cognitive deficits are related to the illness and not undesirable side effect of medication, examination of otherwise stable drug-free patients would be of interest. The period of time between episodes (mania, depression, or seizures), time of testing, hospitalizations, and the presence of psychotic features were not considered in this review. In BD-I patients, the presence of sub-clinical symptoms is common, even in those patients who are rated as euthymic at the time of testing and may have impacted performance overall (3).

Although there is wide variation in the diagnostic criteria of euthymia, our study aimed to control for this by using the HAMD and YMRS as cutoffs; however, longitudinal measurements would have been advantageous to characterize proximity to manic or depressive episodes. Residual mood symptoms are also an important consideration in epilepsy where depression is the most common psychiatric comorbidity (18). In community-based samples, the rates of depression in epilepsy range from 20 to 30% and in hospital samples 20–55% (100, 101). It has been established that depression can cause cognitive impairments, particularly in the domains of attention, psychomotor activity, and memory all of which were relevant to this review (102).

We suggest a strong case may be made for a study comparing neuropsychological tests to assess deficits in BD-I, TLE, and matched HC. In future research, a comprehensive test battery employing tests of attention, executive function, memory, and psychomotor speed, coupled with imaging techniques, should be employed in both disorders relative to HC. This would provide valuable information on the effects of both BD-I and TLE on temporal and other cerebral areas as well as the effects of medication on neuropsychological test parameters. This would also be of value in identifying putative temporal lobe involvement in BD-I.

## Author Contributions

EB, KK, MG, and BT contributed to the design of the project, the analysis and discussion of the results and write up of the paper with KK, MG, and BT contributing their specialist perspective. EB and KK assessed the suitability of papers for inclusion in the manuscript and contributed to the PRISMA review.

## Conflict of Interest Statement

The authors declare that the research was conducted in the absence of any commercial or financial relationships that could be construed as a potential conflict of interest.

## References

[1] Clark L Goodwin GM. State-and trait-related deficits in sustained attention in bipolar disorder. Eur Arch Psychiatry Clin Neurosci (2004) 254(2):61–8. 10.1007/s00406-004-0460-y 15146334

[2] Malhi GS Ivanovski B Szekeres V Olley A. Bipolar disorder: it’s all in your mind? The neuropsychological profile of a biological disorder. Can J Psychiatry (2004) 49(12):813–9. 10.1177/070674370404901204 15679204

[3] Robinson LJ Thompson JM Gallagher P Goswami U Young AH Ferrier IN A meta-analysis of cognitive deficits in euthymic patients with bipolar disorder. J Affect Disord (2006) 93(1–3):105–15. 10.1016/j.jad.2006.02.016 16677713

[4] Torres I Boudreau V Yatham L Neuropsychological functioning in euthymic bipolar disorder: a meta-analysis. Acta Psychiatr Scand Suppl (2007) 116(434):17–26. 10.1111/j.1600-0447.2007.01055.x 17688459

[5] Arts B Jabben N Krabbendam L Van Os J. Meta-analyses of cognitive functioning in euthymic bipolar patients and their first-degree relatives. Psychol Med (2008) 38(06):771–85. 10.1017/S0033291707001675 17922938

[6] Bora E Yucel M Pantelis C. Cognitive endophenotypes of bipolar disorder: a meta-analysis of neuropsychological deficits in euthymic patients and their first-degree relatives. J Affect Disord (2009) 113(1):1–20. 10.1016/j.jad.2008.06.009 18684514

[7] Kurtz MM Gerraty RT. A meta-analytic investigation of neurocognitive deficits in bipolar illness: profile and effects of clinical state. Neuropsychology (2009) 23(5):551. 10.1037/a0016277 19702409 PMC2804472

[8] Mann-Wrobel MC Carreno JT Dickinson D. Meta-analysis of neuropsychological functioning in euthymic bipolar disorder: an update and investigation of moderator variables. Bipolar Disord (2011) 13(4):334–42. 10.1111/j.1399-5618.2011.00935.x 21843273

[9] Bourne C Aydemir Ö Balanzá-Martínez V Bora E Brissos S Cavanagh JTO Neuropsychological testing of cognitive impairment in euthymic bipolar disorder: an individual patient data meta-analysis. Acta Psychiatr Scand (2013) 128(3):149–62. 10.1111/acps.12133 23617548

[10] Krabbendam L Arts B van Os J Aleman A. Cognitive functioning in patients with schizophrenia and bipolar disorder: a quantitative review. Schizophr Res (2005) 80(2–3):137–49. 10.1016/j.schres.2005.08.004 16183257

[11] Deckersbach T Savage CR Reilly-Harrington N Clark L Sachs G Rauch SL Episodic memory impairment in bipolar disorder and obsessive-compulsive disorder: the role of memory strategies. Bipolar Disord (2004) 6:233–44. 10.1111/j.1399-5618.2004.00118.x 15117402

[12] Jones BP Duncan CC Mirsky AF Post RM Theodore WH Neuropsychological profiles in bipolar affective disorder and complex partial seizure disorder. Neuropsychology (1994) 8:55–64. 10.1037/0894-4105.8.1.55

[13] Mazza M Di Nicola M Della Marca G Janiri L Bria P Mazza S. Bipolar disorder and epilepsy: a bidirectional relation? Neurobiological underpinnings, current hypotheses, and future research directions. Neuroscientist (2007) 13(4):392–404. 10.1177/10738584070130041101 17644769

[14] Mula M Marotta AE Monaco F. Epilepsy and bipolar disorders. Expert Rev Neurother (2010) 10(1):13–23. 10.1586/ern.09.139 20021317

[15] Silberman EK Post R Nurnberger J Theodore W Boulenger J Transient sensory, cognitive and affective phenomena in affective illness. A comparison with complex partial epilepsy. Br J Psychiatry (1985) 146(1):81–9. 10.1192/bjp.146.1.81 3978348

[16] Kanner AM Palac S. Depression in epilepsy: a common but often unrecognized comorbid malady. Epilepsy Behav (2000) 1(1):37–51. 10.1006/ebeh.2000.0030 12609126

[17] Bakken IJ Revdal E Nesvåg R Brenner E Knudsen GP Surén P Substance use disorders and psychotic disorders in epilepsy: a population-based registry study. Epilepsy Res (2014) 108(8):1435–43. 10.1016/j.eplepsyres.2014.06.021 25062893

[18] Lyketsos CG Stoline AM Longstreet P Ranen NG Lesser R Fisher R Mania in temporal lobe epilepsy. Cogn Behav Neurol (1993) 6(1):19–25.

[19] Sucksdorff D Brown AS Chudal R Jokiranta-Olkoniemi E Leivonen S Suominen A Parental and comorbid epilepsy in persons with bipolar disorder. J Affect Disord (2015) 188:107–11. 10.1016/j.jad.2015.08.051 26356289 PMC4631649

[20] Bostock ECS Kirkby KC Garry MI Taylor BVM. Comparison of precipitating factors for mania and partial seizures: indicative of shared pathophysiology? J Affect Disord (2015) 183:57–67. 10.1016/j.jad.2015.04.057 26001664

[21] McDonald C Zanelli J Rabe-Hesketh S Ellison-Wright I Sham P Kalidindi S Meta-analysis of magnetic resonance imaging brain morphometry studies in bipolar disorder. Biol Psychiatry (2004) 56(6):411–7. 10.1016/j.biopsych.2004.06.021 15364039

[22] Brambilla P Harenski K Nicoletti M Sassi RB Mallinger AG Frank E MRI investigation of temporal lobe structures in bipolar patients. J Psychiatr Res (2003) 37(4):287–95. 10.1016/S0022-3956(03)00024-4 12765851

[23] Altshuler LL Bartzokis G Grieder T Curran J Jimenez T Leight K An MRI study of temporal lobe structures in men with bipolar disorder or schizophrenia. Biol Psychiatry (2000) 48(2):147–62. 10.1016/S0006-3223(00)00836-2 10903411

[24] Altshuler LL Conrad A Hauser P Ximing L Guze BH Denikoff K Reduction of temporal lobe volume in bipolar disorder: a preliminary report of magnetic resonance imaging. Arch Gen Psychiatry (1991) 48(5):482–3. 10.1001/archpsyc.1991.01810290094018 2021303

[25] Johnstone EC Owens D Crow TJ Frith C Alexandropolis K Bydder G Temporal lobe structure as determined by nuclear magnetic resonance in schizophrenia and bipolar affective disorder. J Neurol Neurosurg Psychiatry (1989) 52(6):736–41. 10.1136/jnnp.52.6.736 2746266 PMC1032025

[26] Swayze VW Andreasen NC Alliger RJ Yuh WT Ehrhardt JC. Subcortical and temporal structures in affective disorder and schizophrenia: a magnetic resonance imaging study. Biol Psychiatry (1992) 31(3):221–40. 10.1016/0006-3223(92)90046-3 1547297

[27] Harvey I Persaud R Ron MA Baker G Murray R. Volumetric MRI measurements in bipolars compared with schizophrenics and healthy controls. Psychol Med (1994) 24(03):689–99. 10.1017/S0033291700027847 7991751

[28] Hauser P Matochik J Altshuler LL Denicoff KD Conrad A Li X MRI-based measurements of temporal lobe and ventricular structures in patients with bipolar I and bipolar II disorders. J Affect Disord (2000) 60(1):25–32. 10.1016/S0165-0327(99)00154-8 10940444

[29] Bernasconi N Andermann F Arnold DL Bernasconi A. Entorhinal cortex MRI assessment in temporal, extratemporal, and idiopathic generalized epilepsy. Epilepsia (2003) 44(8):1070–4. 10.1046/j.1528-1157.2003.64802.x 12887438

[30] Natsume J Bernasconi N Andermann F Bernasconi A. MRI volumetry of the thalamus in temporal, extratemporal, and idiopathic generalized epilepsy. Neurology (2003) 60(8):1296–300. 10.1212/01.WNL.0000058764.34968.C2 12707432

[31] Kuzniecky R Bilir E Gilliam F Faught E Martin R Hugg J. Quantitative MRI in temporal lobe epilepsy evidence for fornix atrophy. Neurology (1999) 53(3):496–496. 10.1212/WNL.53.3.496 10449110

[32] Bernasconi N Duchesne S Janke A Lerch J Collins D Bernasconi A. Whole-brain voxel-based statistical analysis of gray matter and white matter in temporal lobe epilepsy. Neuroimage (2004) 23(2):717–23. 10.1016/j.neuroimage.2004.06.015 15488421

[33] Travers RF Limbic epilepsy. J R Soc Med (1991) 84(8):454. 1909369 10.1177/014107689108400804 PMC1293371

[34] Oyegbile TO Dow C Jones J Bell B Rutecki P Sheth R The nature and course of neuropsychological morbidity in chronic temporal lobe epilepsy. Neurology (2004) 62:1736–42. 10.1212/01.WNL.0000125186.04867.34 15159470

[35] Wang W Liou H Chen C Chiu M Chen T Cheng T Neuropsychological performance and seizure-related risk factors in patients with temporal lobe epilepsy: a retrospective cross-sectional study. Epilepsy Behav (2011) 22:728–34. 10.1016/j.yebeh.2011.08.038 22019015

[36] Moher D Liberati A Tetzlaff J Altman DG Preferred reporting items for systematic reviews and meta-analyses: the PRISMA statement. Ann Intern Med (2009) 151(4):264–9. 10.7326/0003-4819-151-4-200908180-00135 19622511

[37] Altshuler LL Ventura J van Gorp WG Green MF Theberge DC Mintz J. Neurocognitive function in clinically stable men with bipolar I disorder or schizophrenia and normal control subjects. Biol Psychiatry (2004) 56(8):560–9. 10.1016/j.biopsych.2004.08.002 15476685

[38] Bas TO Poyraz CA Bas A Poyraz BC Tosun M. The impact of cognitive impairment, neurological soft signs and subdepressive symptoms on functional outcome in bipolar disorder. J Affect Disord (2015) 174:336–41. 10.1016/j.jad.2014.12.026 25545601

[39] Bora E Hıdıroğlu C Özerdem A Kaçar ÖF Sarısoy G Arslan FC Executive dysfunction and cognitive subgroups in a large sample of euthymic patients with bipolar disorder. Eur Neuropsychopharmacol (2016) 26(8):1338–47. 10.1016/j.euroneuro.2016.04.002 27139077

[40] Chang JS Choi S Ha K Ha TH Cho HS Choi JE Differential pattern of semantic memory organization between bipolar I and II disorders. Prog Neuropsychopharmacol Biol Psychiatry (2011) 35(4):1053–8. 10.1016/j.pnpbp.2011.02.020 21371517

[41] Chou YH Wang SJ Lirng JF Lin CL Yang KC Chen CK Impaired cognition in bipolar I disorder: the roles of the serotonin transporter and brain-derived neurotrophic factor. J Affect Disord (2012) 143(3):131–7. 10.1016/j.jad.2012.05.043 22889524

[42] Deckersbach T McMurrich S Ogutha J Savage CR Sachs G Rauch SL. Characteristics of non-verbal memory impairment in bipolar disorder: the role of encoding strategies. Psychol Med (2004) 34(5):823–32. 10.1017/S0033291703001685 15500303

[43] Dell’Osso B Cinnante C Di Giorgio A Cremaschi L Palazzo MC Cristoffanini M Altered prefrontal cortex activity during working memory task in bipolar disorder: a functional magnetic resonance imaging study in euthymic bipolar I and II patients. J Affect Disord (2015) 184:116–22. 10.1016/j.jad.2015.05.026 26074021

[44] Dittmann S Henning-Fast K Gerber S Seemüller F Riedel M Severus WE Cognitive functioning in euthymic bipolar I and bipolar II patients. Bipolar Disord (2008) 10(8):877–87. 10.1111/j.1399-5618.2008.00640.x 19594503

[45] Dixon T Kravariti E Frith C Murray RM McGuire PK. Effect of symptoms on executive function in bipolar illness. Psychol Med (2004) 34(5):811–21. 10.1017/S0033291703001570 15500302

[46] Doganavşargil-Baysal GO Zehra G Akbaş H Cinemre B Metin O Karaman T. Association of serum homocysteine and methionine levels with cognition and functioning in bipolar disorder. Turk Psikiyatri Derg (2013) 24(1):6–14. 23446535

[47] Doruk A Yazihan NT Balikci A Erdem M Bolu A Ates MA Cognitive functions in bipolar manic, depressed and remission episodes. Klinik Psikofarmakoloji Bulteni (2014) 24(1):59–68. 10.5455/bcp.20130506122642

[48] Fistikci N Canturk G Saatcioglu O Erten E Keyvan A Turan N Executive functions and thyroid volumes in bipolar patients on lithium treatment. Afr J Psychiatry (South Africa) (2014) 17(6):145 10.4172/Psychiatry.1000145

[49] Frangou S Haldane M Roddy D Kumari V. Evidence for deficit in tasks of ventral, but not dorsal, prefrontal executive function as an endophenotypic marker for bipolar disorder. Biol Psychiatry (2005) 58(10):838–9. 10.1016/j.biopsych.2005.05.020 16043135

[50] Hsiao Y-L Wu Y-S Wu JY-W Hsu M-H Chen H-C Lee S-Y Neuropsychological functions in patients with bipolar I and bipolar II disorder. Bipolar Disord (2009) 11(5):547–54. 10.1111/j.1399-5618.2009.00723.x 19624394

[51] Martino DJ Strejilevich SA Torralva T Manes F. Decision making in euthymic bipolar I and bipolar II disorders. Psychol Med (2011) 41(6):1319–27. 10.1017/S0033291710001832 20860871

[52] Muralidharan K Kozicky JM Bücker J Silveira LE Torres IJ Yatham LN. Are cognitive deficits similar in remitted early bipolar I disorder patients treated with lithium or valproate? Data from the STOP-EM study. Eur Neuropsychopharmacol (2015) 25(2):223–30. 10.1016/j.euroneuro.2014.09.005 25261261

[53] Muralidharan K Torres IJ Silveira LE Kozicky JM Bücker J Fernando N Impact of depressive episodes on cognitive deficits in early bipolar disorder: data from the systematic treatment optimization programme for early mania (STOP-EM). Br J Psychiatry (2014) 205(1):36–43. 10.1192/bjp.bp.113.135525 24764544

[54] Normala I Abdul Hamid AR Azlin B Nik Ruzyanei NJ Hazli Z Shah SA. Executive function and attention span in euthymic patients with bipolar 1 disorder. Med J Malaysia (2010) 65(3):192–6. 21939168

[55] Pattanayak RD Sagar R Mehta M. Neuropsychological performance in euthymic Indian patients with bipolar disorder type I: correlation between quality of life and global functioning. Psychiatry Clin Neurosci (2012) 66(7):553–63. 10.1111/j.1440-1819.2012.02400.x 23252921

[56] Radwan DN Cognitive impairment in Egyptian euthymic patients with bipolar I disorder compared with controls. Middle East Curr Psychiatry (2013) 20(4):197–204. 10.1097/01.XME.0000433325.69290.c9

[57] Sepede G De Berardis D Campanella D Perrucci MG Ferretti A Serroni N Impaired sustained attention in euthymic bipolar disorder patients and non-affected relatives: an fMRI study. Bipolar Disord (2012) 14(7):764–79. 10.1111/bdi.12007 23036083

[58] Trivedi JK Dhyani M Sharma S Sinha PK Singh AP Tandon R. Cognitive functions in euthymic state of bipolar disorder: an Indian study. Cogn Neuropsychiatry (2008) 13(2):135–47. 10.1080/13546800801897346 18302026

[59] Trivedi JK Goel D Sharma S Singh AP Sinha PK Tandon R. Cognitive functions in stable schizophrenia and euthymic state of bipolar disorder. Ind J Med Res (2007) 126(5):433–9. 18160747

[60] Yates DB Dittmann S Kapczinski F Trentini CM. Cognitive abilities and clinical variables in bipolar I depressed and euthymic patients and controls. J Psychiatr Res (2011) 45(4):495–504. 10.1016/j.jpsychires.2010.09.006 20951385

[61] Zubieta J-K Huguelet P O’Neil RL Giordani BJ Cognitive function in euthymic bipolar I disorder. Psychiatry Res (2001) 102(1):9–20. 10.1016/S0165-1781(01)00242-6 11368835

[62] Baxendale S Thompson P. Defining meaningful postoperative change in epilepsy surgery patients: measuring the unmeasurable? Epilepsy Behav (2005) 6(2):207–11. 10.1016/j.yebeh.2004.12.009 15710306

[63] Baxendale S Thompson PJ Sander JW. Neuropsychological outcomes in epilepsy surgery patients with unilateral hippocampal sclerosis and good preoperative memory function. Epilepsia (2013) 54(9):131–4. 10.1111/epi.12319 23875960

[64] Berenbaum SA Baxter L Seidenberg M Hermann B. Role of the hippocampus in sex differences in verbal memory: memory outcome following left anterior temporal lobectomy. Neuropsychology (1997) 11(4):585–91. 10.1037/0894-4105.11.4.585 9345702

[65] Bjørnæs H Stabell KE Røste GK Bakke SJ. Changes in verbal and nonverbal memory following anterior temporal lobe surgery for refractory seizures: effects of sex and laterality. Epilepsy Behav (2005) 6(1):71–84. 10.1016/j.yebeh.2004.10.011 15652737

[66] Chelune GJ Naugle RI Lüders H Awad IA. Prediction of cognitive change as a function of preoperative ability status among temporal lobectomy patients seen at 6-month follow-up. Neurology (1991) 41(3):399–404. 10.1212/WNL.41.3.399 2006008

[67] Chiaravalloti ND Tulsky DS Glosser G. Validation of the WMS-III facial memory subtest with the graduate hospital facial memory test in a sample of right and left anterior temporal lobectomy patients. J Clin Exp Neuropsychol (2004) 26(4):484–97. 10.1080/13803390490496623 15512936

[68] Chiaravalloti ND Glosser G. Material-specific memory changes following anterior temporal lobectomy as predicted by the intracarotid amobarbital test. Epilepsia (2001) 42(7):902–11. 10.1046/j.1528-1157.2001.02500.x 11488891

[69] Fernandes DA Yasuda CL Lopes TM Enrico G Alessio A Tedeschi H Long-term postoperative atrophy of contralateral hippocampus and cognitive function in unilateral refractory MTLE with unilateral hippocampal sclerosis. Epilepsy Behav (2014) 36:108–14. 10.1016/j.yebeh.2014.04.028 24907496

[70] Giovagnoli AR Casazza M Ciceri E Avanzini G Broggi G. Preserved memory in temporal lobe epilepsy patients after surgery for low-grade tumour. A pilot study. Neurol Sci (2007) 28(5):251–8. 10.1007/s10072-007-0831-z 17972039

[71] Gleissner U Helmstaedter C Schramm J Elger CE. Memory outcome after selective amygdalohippocampectomy in patients with temporal lobe epilepsy: one-year follow-up. Epilepsia (2004) 45(8):960–2. 10.1111/j.0013-9580.2004.42203.x 15270763

[72] Glosser G Salvucci AE Chiaravalloti ND. Naming and recognizing famous faces in temporal lobe epilepsy. Neurology (2003) 61(1):81–6. 10.1212/01.WNL.0000073621.18013.E1 12847161

[73] Helmstaedter C Grunwald T Lehnertz K Gleissner U Elger CE. Differential involvement of left temporolateral and temporomesial structures in verbal declarative learning and memory: evidence from temporal lobe epilepsy. Brain Cogn (1997) 35(1):110–31. 10.1006/brcg.1997.0930 9339305

[74] Hermann BP Wyler AR. Effects of anterior temporal lobectomy on language function: a controlled study. Ann Neurol (1988) 23(6):585–8. 10.1002/ana.410230610 3408239

[75] Hermann BP Wyler AR Bush AJ Tabatabai FR. Differential effects of left and right anterior temporal lobectomy on verbal learning and memory performance. Epilepsia (1992) 33(2):289–97. 10.1111/j.1528-1157.1992.tb02318.x 1547758

[76] Hermann BP Wyler AR Somes G Dohan FC Jr Berry AD III Clement L. Declarative memory following anterior temporal lobectomy in humans. Behav Neurosci (1994) 108(1):3–10. 10.1037/0735-7044.108.1.3 8192849

[77] Köylü B Walser G Ischebeck A Ortler M Benke T. Functional imaging of semantic memory predicts postoperative episodic memory functions in chronic temporal lobe epilepsy. Brain Res (2008) 1223:73–81. 10.1016/j.brainres.2008.05.075 18599025

[78] Lee T Mackenzie RA Walker AJ Matheson JM Sachdev P. Effects of left temporal lobectomy and amygdalohippocampectomy on memory. J Clin Neurosci (1997) 4(3):314–9. 10.1016/S0967-5868(97)90098-9 18638976

[79] Loring DW Lee GP Martin RC Meador KJ Material-specific learning in patients with partial complex seizures of temporal lobe origin: convergent validation of memory constructs. J Epilepsy (1988) 1(2):53–9. 10.1016/s0896-6974(88)80057-4

[80] Malikova H Kramska L Vojtech Z Lukavsky J Liscak R. Stereotactic radiofrequency amygdalohippocampectomy: two years of good neuropsychological outcomes. Epilepsy Res (2013) 106(3):423–32. 10.1016/j.eplepsyres.2013.07.009 23968819

[81] Morino M Ichinose T Uda T Kondo K Ohfuji S Ohata K. Memory outcome following transsylvian selective amygdalohippocampectomy in 62 patients with hippocampal sclerosis. J Neurosurg (2009) 110(6):1164–9. 10.3171/2008.9.jns08247 19119880

[82] Quigg M Broshek DK Barbaro NM Ward MM Laxer KD Yan G Neuropsychological outcomes after gamma knife radiosurgery for mesial temporal lobe epilepsy: a prospective multicenter study. Epilepsia (2011) 52(5):909–16. 10.1111/j.1528-1167.2011.02987.x 21426323 PMC3519361

[83] Seidenberg M Hermann BP Dohan FC Jr Wyler AR Perrine A Schoenfeld J. Hippocampal sclerosis and verbal encoding ability following anterior temporal lobectomy. Neuropsychologia (1996) 34(7):699–708. 10.1016/0028-3932(95)00146-8 8783221

[84] Selwa LM Berent S Giordani B Henry TR Buchtel HA Ross DA. Serial cognitive testing in temporal lobe epilepsy: longitudinal changes with medical and surgical therapies. Epilepsia (1994) 35(4):743–9. 10.1111/j.1528-1157.1994.tb02505.x 8082616

[85] Shamim S Wiggs E Heiss J Sato S Liew C Solomon J Temporal lobectomy: resection volume, neuropsychological effects, and seizure outcome. Epilepsy Behav (2009) 16(2):311–4. 10.1016/j.yebeh.2009.07.040 19703792 PMC2785019

[86] Stretton J Sidhu MK Winston GP Bartlett P McEvoy AW Symms MR Working memory network plasticity after anterior temporal lobe resection: a longitudinal functional magnetic resonance imaging study. Brain (2014) 137(5):1439–53. 10.1093/brain/awu061 24691395 PMC3999723

[87] Tisser L Palmini A Paglioli E Portuguez M Azambuja N da Costa JC Pre and post-operative Wisconsin card sorting test performance in patients with temporal lobe epilepsy due to hippocampal sclerosis. Dement Neuropsychol (2007) 1(2):173–80. 10.1590/s1980-57642008dn10200010 PMC5619566 29213385

[88] Trenerry MR Jack CR Wisconsin card sorting test performance before and after temporal lobectomy. J Epilepsy (1994) 7(4):313–7. 10.1016/0896-6974(94)90062-0

[89] Trenerry MR Jack CR Jr Ivnik RJ Sharbrough FW Cascino GD Hirschorn KA MRI hippocampal volumes and memory function before and after temporal lobectomy. Neurology (1993) 43(9):1800–5. 10.1212/WNL.43.9.1800 8414035

[90] von Rhein B Nelles M Urbach H Von Lehe M Schramm J Helmstaedter C. Neuropsychological outcome after selective amygdalohippocampectomy: subtemporal versus transsylvian approach. J Neurol Neurosurg Psychiatry (2012) 83(9):887–93. 10.1136/jnnp-2011-302025 22773854

[91] d’Esposito M Aguirre G Zarahn E Ballard D Shin R Lease J Functional MRI studies of spatial and nonspatial working memory. Brain Res Cogn Brain Res (1998) 7(1):1–13. 10.1016/S0926-6410(98)00004-4 9714705

[92] Lee T Yip JT Jones-Gotman M. Memory deficits after resection from left or right anterior temporal lobe in humans: a meta-analytic review. Epilepsia (2002) 43(3):283–91. 10.1046/j.1528-1157.2002.09901.x 11906514

[93] Vaz SAM. Nonverbal memory functioning following right anterior temporal lobectomy: a meta-analytic review. Seizure (2004) 13(7):446–52. 10.1016/j.seizure.2003.12.004 15324819

[94] Devinsky O. The myth of silent cortex and the morbidity of epileptogenic tissue: implications for temporal lobectomy. Epilepsy Behav (2005) 7(3):383–9. 10.1016/j.yebeh.2005.07.020 16198151

[95] Lieb JP Dasheiff RM Engel J. Role of the frontal lobes in the propagation of mesial temporal lobe seizures. Epilepsia (1991) 32(6):822–37. 10.1111/j.1528-1157.1991.tb05539.x 1743154

[96] Rusnáková Š Daniel P Chládek J Jurák P Rektor I The executive functions in frontal and temporal lobes: a flanker task intracerebral recording study. J Clin Neurophysiol (2011) 28(1):30–5. 10.1097/WNP.0b013e31820512d4 21221007

[97] Pachet AK Wisniewski AM. The effects of lithium on cognition: an updated review. Psychopharmacology (2003) 170(3):225–34. 10.1007/s00213-003-1592-x 14504681

[98] Alda M McKinnon M Blagdon R Garnham J MacLellan S O’donovan C Methylene blue treatment for residual symptoms of bipolar disorder: randomised crossover study. Br J Psychiatry (2016) 210(1):54–60. 10.1192/bjp.bp.115.173930 27284082

[99] Furian AF Fighera MR Oliveira MS de Oliveira Ferreira AP Fiorenza NG de Carvalho Myskiw J Methylene blue prevents methylmalonate-induced seizures and oxidative damage in rat striatum. Neurochem Int (2007) 50(1):164–71. 10.1016/j.neuint.2006.07.012 16963161

[100] Blum D Reed M Metz A Prevalence of major affective disorders and manic/hypomanic symptoms in persons with epilepsy: a community survey. Neurology (2002) 58(7):175.

[101] Kanner AM Balabanov A. Depression and epilepsy how closely related are they? Neurology (2002) 58(8 Suppl 5):S27–39. 10.1212/WNL.58.8_suppl_5.S27 11971130

[102] Gallassi R Morreale A Pagni P The relationship between depression and cognition. Arch Gerontol Geriatr (2001) 33:163–71. 10.1016/S0167-4943(01)00136-4 11431061

